# Deciphering the Great Imitator: Syphilis and Neurosyphilis

**DOI:** 10.7759/cureus.54563

**Published:** 2024-02-20

**Authors:** Alisa Zezetko, Matthew Stallings, Irene Pastis

**Affiliations:** 1 Psychiatry, East Carolina University Health Medical Center, Greenville, USA; 2 Psychiatry, Brody School of Medicine, East Carolina University, Greenville, USA

**Keywords:** fta-abs, vdrl, treponema pallidum, neurosyphilis, syphilis

## Abstract

Syphilis is an infectious disease caused by *Treponema pallidum*. Often known as the “great imitator,” it has periods of active disease and periods of latency. Serologic syphilis testing can be divided into treponemal and non-treponemal tests, and multiple tests are required to prove infection. Standardized algorithms exist for syphilis testing and diagnosis. Neurosyphilis, which is often the result of the progression of untreated syphilis, can be life-threatening and requires intravenous antibiotics. Despite the significant challenge of diagnosing and treating neurosyphilis, there are no standardized testing algorithms available. Typically, the cerebral spinal fluid (CSF) venereal disease research laboratory (VDRL) test is considered the gold standard despite low sensitivity. The CSF fluorescent treponemal antibody absorption (FTA-ABS) test is more sensitive despite being less specific and is often the better testing option. This case illustrates a patient with a clinical presentation strongly suggestive of neurosyphilis despite negative initial lab testing and argues for the emergence of a standardized algorithm to guide clinicians.

## Introduction

Since reaching a historic low in 2000 and 2001, the rate of primary and secondary syphilis infections has increased almost every year. From 2020 to 2021, the rate increased by 28.6%. The rate of congenital syphilis has also increased every year since 2013 with a 30.5% increase from 2020 to 2021 [1]. Often known as the “great imitator,” syphilis has periods of active and latent disease. Primary syphilis starts 10 to 90 days after exposure and results in a painless indurated ulcer or chancre at the site of inoculation. In three to six weeks, these lesions disappear without treatment. Secondary syphilis starts two to eight weeks after the chancre disappears and can have multiple different systemic manifestations. Dermatologic manifestations are usually highly contagious during this phase, and lesions contain high loads of *Treponema pallidum* spirochetes. Without treatment, primary or secondary syphilis can progress to either an early latent phase that lasts one year or a late latent phase that lasts more than one year. During either latent phase, serologic tests are positive, but there are no clinical manifestations. Tertiary syphilis develops months to years after the initial infection. It can manifest as cardiovascular syphilis with aortic aneurysms and aortic valvulopathy, gummatous syphilis with infiltration and destruction of organs by the bacterium, or neurosyphilis [2]. The term neurosyphilis refers to an infection of the central nervous system (CNS) and can occur at any time after infection. CNS infection can resolve spontaneously, be asymptomatic, or proceed to transient or persistent meningitis [3,4]. There are five types of neurosyphilis: asymptomatic, meningeal, meningovascular, general paresis, and tabes dorsalis [5-7].

Early neurosyphilis can be asymptomatic, meningeal, or meningovascular. Asymptomatic neurosyphilis is the most common, and patients are often unaware that they are affected. It is defined by the presence of serological evidence of syphilis, cerebrospinal fluid (CSF) abnormalities, and no neurological symptoms. Meningeal neurosyphilis results from diffuse inflammation of the meninges and can lead to headache, nausea, vomiting, neck stiffness, photophobia, cranial nerve deficits, and even seizures. Meningovascular neurosyphilis is defined as the inflammation of the meninges in addition to endarteritis causing thrombosis and infarction of cerebral tissue. Symptoms include headache, nausea, vomiting, and vertigo from meningeal inflammation and cerebral vascular deficits related to the site of thrombosis and corresponding cerebral functions. In addition, spinal cord vessels may also be affected leading to meningomyelitis, spastic weakness, sensory loss, and muscular atrophy [5-7].

Late neurosyphilis is divided into general paresis and tabes dorsalis. General paresis usually results from chronic meningoencephalitis that leads to cerebral atrophy. Manifestations can be divided into early and late symptoms that can occur insidiously or suddenly. Early symptoms include mood disturbances, such as irritability, personality changes, changes in sleep habits, and forgetfulness; late symptoms include labile mood, memory and judgment impairment, confusion, delusions, and seizures. Depression, delirium, mania, and psychosis may also occur. This stage is usually accompanied by pupillary abnormalities, dysarthria, and tremors. Tabes dorsalis results from degeneration of the posterior or dorsal columns and roots of the spinal cord, and patients at this stage typically have ataxia, lightning pains, bladder dysfunction, paresthesias, and vision changes. Argyll Robertson pupils, ocular palsies, diminished reflexes, vibratory and proprioceptive impairments, and Charcot joints are also found during this stage. Untreated neurosyphilis can result in paralysis, dementia, and death [5-7].

Serologic syphilis testing can be divided into treponemal and non-treponemal tests. Treponemal testing includes Captia Syphilis-G enzyme immunoassay (EIA) and Serodia Treponema pallidum particle agglutination (TP-PA) testing; these detect syphilis-specific antibodies from different species and subspecies of *Treponema*. They are positive for life after exposure and cannot distinguish between old and new infections and treated and untreated infections. Non-treponemal tests include rapid plasma regain (RPR) and venereal disease research laboratory (VDRL) tests that detect antibodies to cellular components released during syphilis-induced tissue damage. These are reported in titers and are often used to monitor response to treatment. Non-treponemal tests are less sensitive, as, with or without treatment, titers will decline over time unless an individual is re-infected. They are also less specific and can be high in autoimmune diseases and acute febrile illnesses. Treponemal tests are consequently usually used first in testing for syphilis, as they are more sensitive and specific despite their lack of clarity involving treatment status and stage of infection [8].

There is no standardized testing for neurosyphilis, and diagnosis is often challenging relying on clinical presentation and CSF analysis findings. CSF analysis is generally not recommended if patients do not have neurological symptoms; however, if a patient is diagnosed with tertiary syphilis, then CSF analysis should be done prior to treatment to ensure the initiation of a neurosyphilis treatment regimen if necessary. CSF VDRL is highly specific and generally accepted as the diagnostic test of choice for neurosyphilis. CSF RPR is not recommended because of lower sensitivity. CSF fluorescent treponemal antibody test absorption tests (FTA-ABS) are highly sensitive but nonspecific, so they can be used to rule out neurosyphilis when the pretest probability is moderate to low. CSF analysis usually reveals pleocytosis with a white blood cell count of 20 cells per microliter or more and elevated protein levels. CSF analysis is sensitive but not specific for infectious and noninfectious causes. This test can also be convoluted in patients who have human immunodeficiency virus (HIV), as they are likely to have pleocytosis. Neuroimaging can be helpful, but findings are generally nonspecific. The most common findings are frontal and temporoparietal atrophy. Nonspecific white matter lesions are frequently found in patients with tabes dorsalis. Patients with meningovascular disease often have changes consistent with infarction [5-7]. 

Parenteral penicillin G is required for all stages of syphilis. Primary, secondary, or early latent syphilis is treated with a single dose of intramuscular benzathine penicillin G 2.4 million units. Tertiary and latent syphilis and HIV-infected patients require weekly benzathine penicillin G 2.4 million units intramuscularly for three weeks [9,10]. In neurosyphilis, the recommended treatment is penicillin G 18 to 24 million units intravenously daily for 10 to 14 days or penicillin G 24 million units intravenously continuously for 10 to 14 days [5,11]. After treatment, tests of cure with VDRL or RPR are recommended; in early syphilis, testing should be done at three, six, and 12 months, and in late syphilis and neurosyphilis, testing should be done every six months until it is negative. A four-fold decline in titers of RPR or VDRL usually indicates successful treatment [12]. This case emphasizes the importance of creating standardized testing algorithms for neurosyphilis that can guide clinicians in the diagnosis and treatment of this potentially fatal but relatively easily treatable infection. 

This article was previously presented as a meeting abstract at the 2023 East Carolina University (ECU) Health Graduate Medical Education (GME) Research Day Meeting on May 18, 2023.

## Case presentation

A 71-year-old female was admitted to the hospital from her assisted living facility with hyperglycemia and personality change, delusions, and hallucinations for the last month. Concerns for dementia began as early as five years ago, and she had a diagnosis of mild neurocognitive disorder secondary to vascular dementia. She had come to her assisted living facility one year ago when she was very high functioning, but she has been steadily declining since then. She also had a history of previous cerebrovascular accidents (CVA) and extensive coronary artery disease (CAD) requiring surgical intervention. Past medical history was also significant for heart failure, stage four chronic kidney disease, type two diabetes, hypertension, hyperlipidemia, and osteoarthritis. 

**Figure 1:**
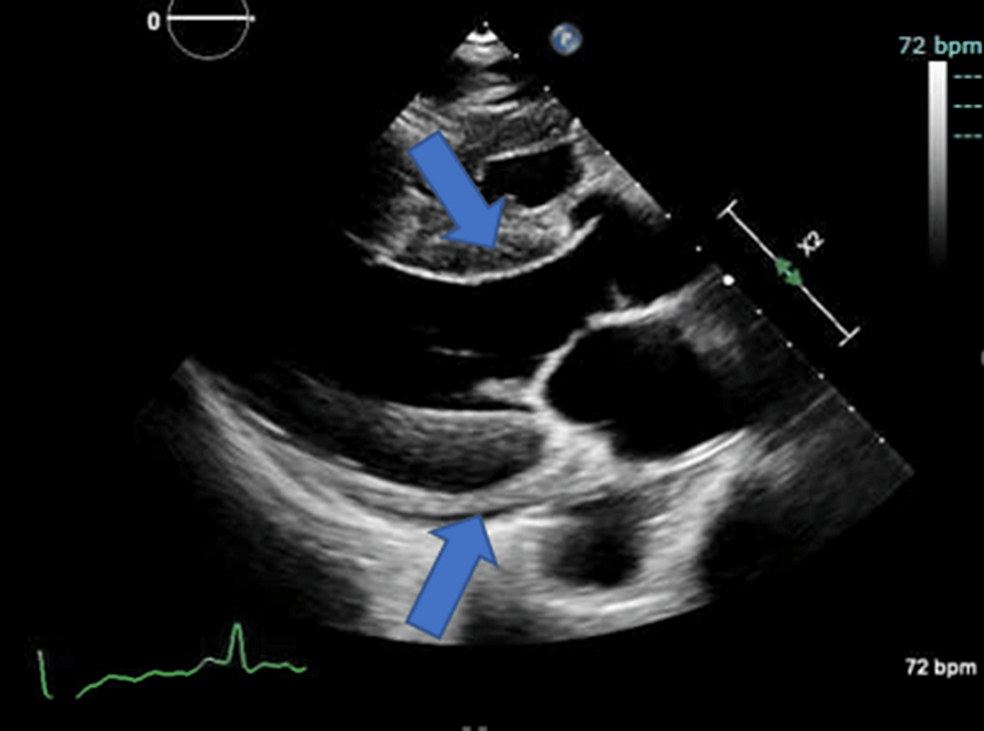
Echocardiogram showing mild global hypokinesis of the left ventricle, mild concentric left ventricular hypertrophy, and hyperechoic myocardium with no major valve insufficiencies.

**Figure 2:**
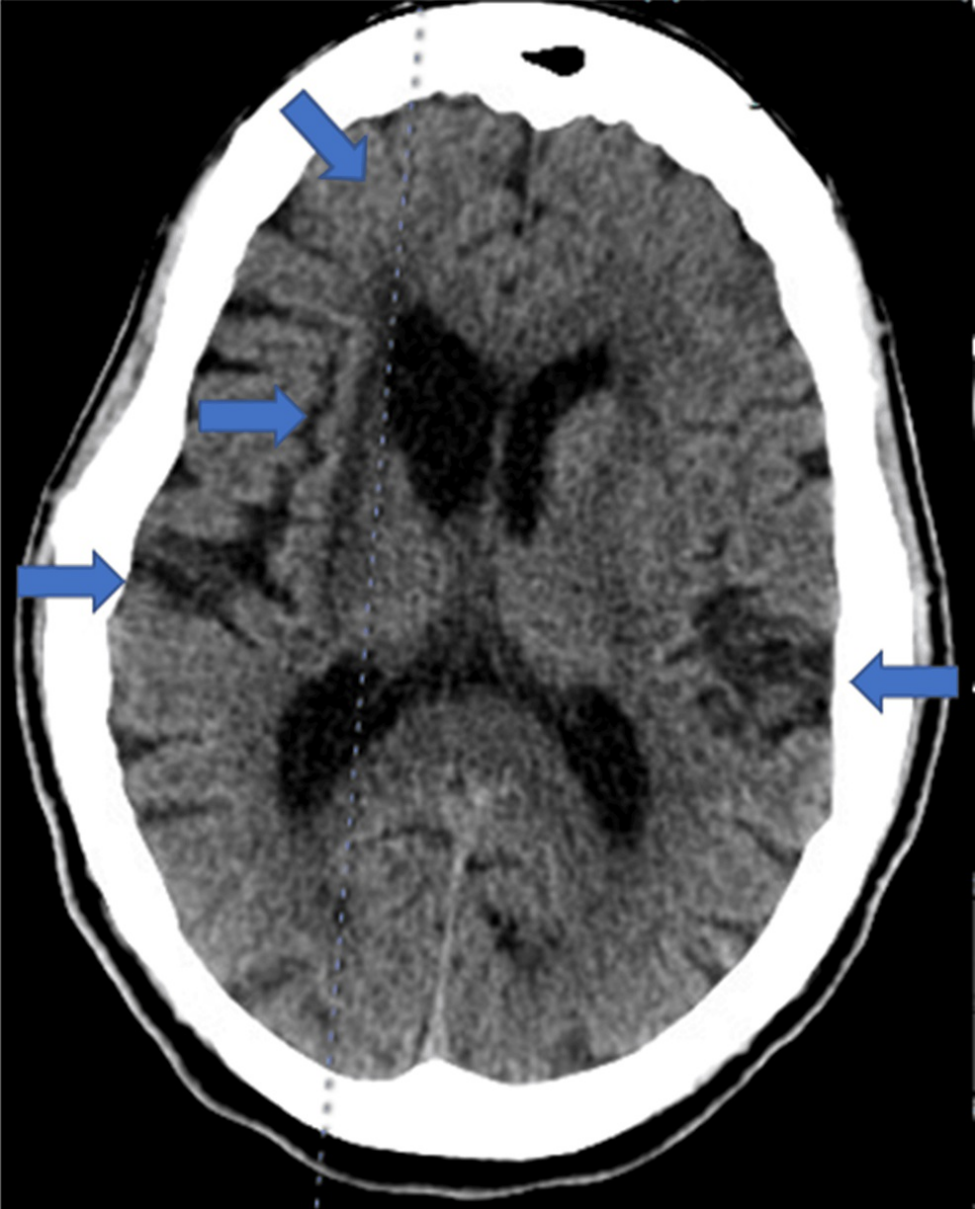
Computed tomography without intravenous contrast showing chronic changes included an infarct involving the right lateral basal ganglia, sub-insular white matter, and right frontal periventricular white matter with associated volume loss with ex vacuo dilatation of the frontal horn of the right lateral ventricle. There are also scattered areas of hypodensity within the white matter of both cerebral hemispheres that are nonspecific.

This was her second admission and third emergency department (ED) visit in the last week. Testing from a recent three-day admission for altered mental status and similar delusions revealed unremarkable ammonia, thyroid-stimulating hormone (TSH), and respiratory panel. Troponins were elevated at 0.10 ng/mL, and brain natriuretic peptide (BNP) was high at 127 pg/mL (Table 1). An echocardiogram (ECHO) revealed mild global hypokinesis of the left ventricle, mild concentric left ventricular hypertrophy, and hyperechoic myocardium with no major valve insufficiencies (Figure 1). Head computed tomography (CT) without intravenous contrast revealed no acute intracranial abnormality. Chronic changes included an infarct involving the right lateral basal ganglia, sub-insular white matter, and right frontal periventricular white matter with associated volume loss with ex vacuo dilatation of the frontal horn of the right lateral ventricle. In addition, there were scattered areas of hypodensity within the white matter of both cerebral hemispheres that were nonspecific (Figure 2). During that admission, the patient was thought to be suffering from delirium secondary to Benadryl use and constipation. Her Benadryl was discontinued, laxatives were added to her medication regimen, and she was discharged after having a bowel movement. Still, her delusions continued even on the day of discharge when her delirium was considered to be resolved. She was brought to the ED again a few days later for continued delusions but was discharged again after the lab workup was unremarkable. All other medical, family, and psychosocial histories were non-contributory.

**Table 1:** Relevant previous hospital admission laboratory results TSH: thyroid-stimulating hormone; BNP: B-type natriuretic peptide; PCR: polymerase chain reaction

Test/units	Result	Reference range
Ammonia (umol/L)	30	18-72
TSH (uIU/mL)	1.36	0.35-4.94
Troponin I (ng/mL)	0.10	≤0.03
BNP (pg/mL)	127	≤100
Influenza A by PCR	Negative	Negative
Influenza B by PCR	Negative	Negative
Respiratory syncytial virus	Negative	Negative

On this admission, a physical exam revealed a mild left-sided facial droop and slurred speech thought to be a sequelae of her stroke. The patient was also confused and could not answer questions appropriately. She perseverated on her delusions. She had poor hearing, and her gait was unsteady. Throughout her stay, she was noted to have intermittent flapping of her hands in repetitive motions. A gynecological exam was not completed, and no remarkable skin findings were noted. Otherwise, the physical exam was unremarkable.

**Figure 3:**
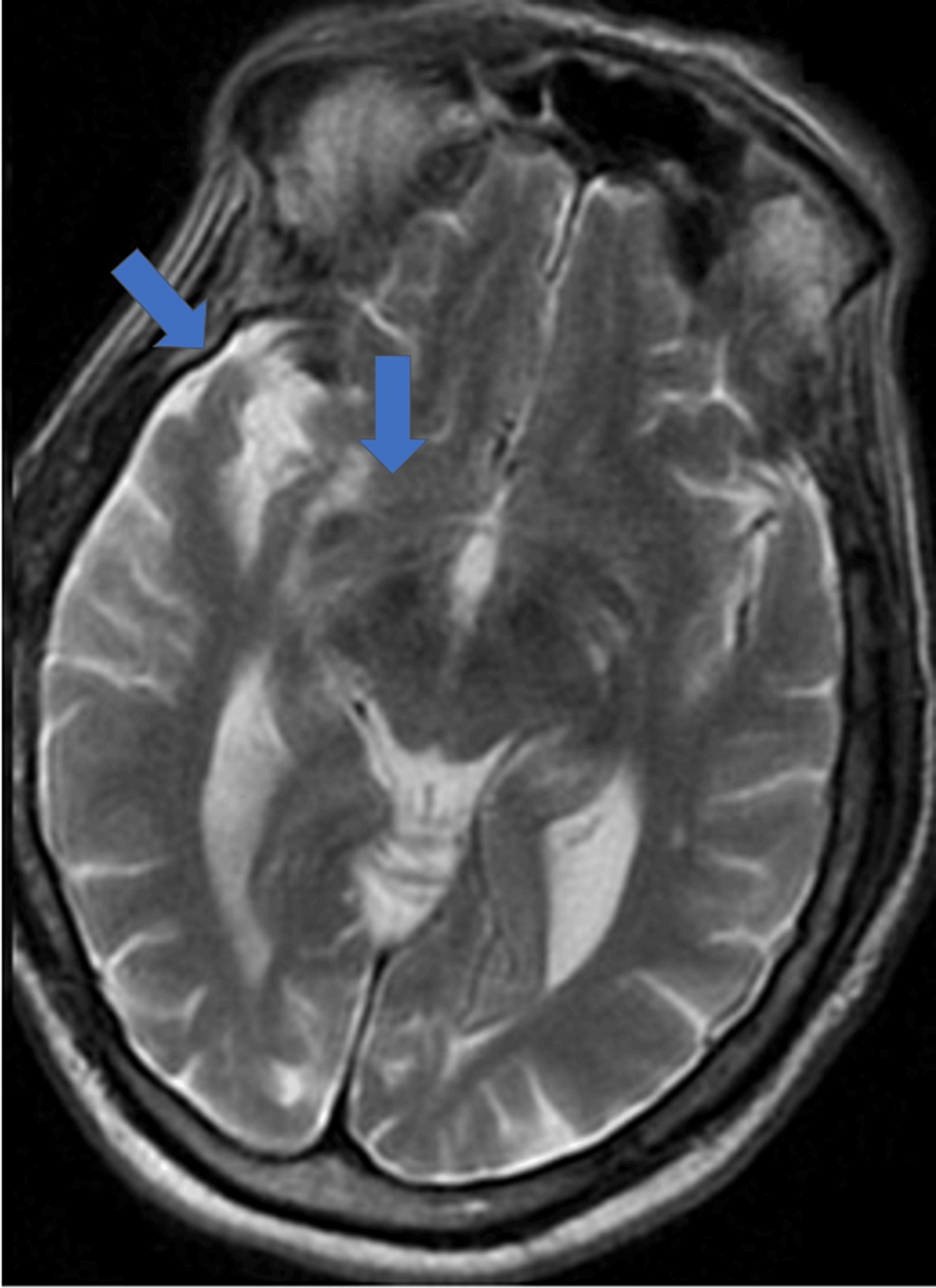
Magnetic resonance imaging without intravenous contrast was limited due to motion and was negative for stroke, mass, and intracranial bleeding. It revealed the same chronic infarct involving the lateral right basal ganglia as previously noted on head CT one week earlier.

Initial blood analysis included high glucose of 254 mg/dL with a slight anion gap of 14 mEq/L and elevated creatinine of 1.82 mg/dL, which was around the patient's usual baseline per chart review. Otherwise, complete blood count (CBC) and complete metabolic panel (CMP) were non-contributory. Vitamin B12 was within normal limits, and vitamin D was low at 28.5 ng/mL. Urinalysis was negative for leukocyte esterase or nitrites. Urine culture revealed no growth. Urine drug screen (UDS) was negative. On admission, further labs were ordered. Folate was within normal limits. HIV studies were negative (Table 2). Syphilis immunoglobulin G (IGG) antibodies with reflex enzyme immunoassay returned reactive triggering rapid plasma reagin (RPR) with reflex to titer. RPR was negative, triggering syphilis antibody TP-PA testing, which was positive (Table 3). Magnetic resonance imaging (MRI) without intravenous contrast was limited due to motion and was negative for stroke, mass, and intracranial bleeding. It revealed the same chronic intracranial findings previously noted on head CT one week earlier (Figure 3).

**Table 2:** Initial blood analysis on presentation and further laboratory results CBC: complete blood count; WBC: white blood cells; RBC: red blood cells; MCV: mean corpuscular volume; RDW: red blood cell distribution width; BMP: basic metabolic panel; BUN: blood urea nitrogen; GFR: glomerular filtration rate; pH: potential hydrogen; HIV: human immunodeficiency virus; MDMA: methyl​enedioxy​methamphetamine; ID: identification; AB/AG: antibody/antigen; AG/AB: antigen/antibody

Test/units	Results	Reference range
CBC		
WBC (k/uL)	5.95	4.50–11.00
RBC (M/uL)	3.46	3.80–5.20
Hemoglobin (g/dL)	10.0	12.0–16.0
Hematocrit (%)	30.5	35.0–47.0
MCV (fL)	88.2	80.0–100.0
RDW (%)	13.7	11.5–14.5
Platelet (k/uL)	204	150–440
Mean platelet volume (fL)	11.3	7.4–10.6
Neutrophil (%)	64	NA
Lymphocyte (%)	27	NA
Monocyte (%)	9	NA
Eosinophil (%)	0	NA
Basophil (%)	0	NA
Immature granulocytes (%)	0	NA
BMP		
Sodium (mEq/L)	138	136–145
Potassium (mEq/L)	3.9	3.5–5.1
Chloride (mEq/L)	101	98–107
Bicarbonate (TCO2) (mEq/L)	23	23–31
Anion gap (calculated) (mEq/L)	14	4–12
Glucose (mg/dL)	254	70–105
BUN (mg/dL)	31	10–20
Creatinine (mg/dL)	1.82	0.57–1.11
GFR (mL/Min/1.73 m2)	29	≥59
Calcium (mg/dL)	9.5	8.4–10.2
Phosphorus (mg/dL)	3.2	2.3–4.7
Magnesium (mg/dL)	1.8	1.6–2.6
Nutrition		
Folate (ng/mL)	14.3	>5.4
Vitamin B12 (pg/mL)	745	213–816
Vitamin D, total (ng/mL)	28.5	30.0–100.0
Urinalysis, urine culture, urine drug screen		
Appearance, urine	Clear	Clear
Color, urine	Straw	Colorless, yellow, or straw
pH, urine	6.5	5.0–8.0
Specific gravity, urine	1.013	1.005–1.030
Protein, urine	1+	Negative
Glucose, urine	3+	Negative
Ketones, urine	Trace	Negative
Bilirubin, urine	Negative	Negative
Hemoglobin, urine	1+	Negative
Leukocyte esterase, urine	Negative	Negative
Nitrites, urine	Negative	Negative
Urobilinogen, urine	Normal	Normal
WBC, urine (/HPF)	1	0
RBC, urine (/HPF)	2	0
Bacteria, urine	Present	None seen
Squamous epithelial cells (/HPF)	None seen	None seen
Non-squamous epithelial cells, urine (/HPF)	Few	None
Hyaline casts, urine (/LPF)	Present	None
Urine culture	No growth	No growth
Amphetamine, urine	Below detection limit	Below detection limit
Barbiturates, urine	Below detection limit	Below detection limit
Benzodiazepines, urine	Below detection limit	Below detection limit
Cannabinoids, urine	Below detection limit	Below detection limit
Cocaine, urine	Below detection limit	Below detection limit
Opiates, urine	Below detection limit	Below detection limit
Phencyclidine, urine	Below detection limit	Below detection limit
MDMA screen, urine	Below detection limit	Below detection limit
Fentanyl screen, urine	Below detection limit	Below detection limit
Methadone, urine	Below detection limit	Below detection limit
Blood ID		
HIV AB/AG combined ASSAY	Negative	Negative
HIV AG/AB combo	Negative	Negative

**Table 3:** Syphilis test results IgG: immunoglobulin G; EIA: enzyme immunoassay; RPR: rapid plasma reagin; Ab: antibody; TP-PA: Serodia Treponema pallidum particle  agglutination; S: serum

Test/Units	Result	Reference range
Syphilis IgG w/ reflex, EIA, S	Reactive	Nonreactive
RPR Screen w/ reflex to titer, S	Negative	Negative
Syphilis Ab by TP-PA, S	Positive	Negative

A lumbar puncture was performed. CSF analysis revealed no pleocytosis with a total nucleated cell count of 1/uL and a slightly elevated protein count of 48 mg/dL. Glucose was 92 mg/dL, and red blood cells (RBC) were 2/uL. Other findings were within normal limits. The CSF culture revealed no growth after three days, the gram stain revealed no white blood cells (WBCs), and no organisms were seen. Herpes simplex virus (HSV) 1 and 2 testing was negative, and CSF VDRL was also negative (Table 4).

**Table 4:** CSF findings CSF: cerebrospinal fluid; WBC: white blood cells; PCR: polymerase chain reaction; VDRL: venereal disease research laboratory

Test/units	Result	Reference range
Volume, CSF (mL)	13	NA
Color, CSF	Colorless	Colorless
Turbidity, CSF	Clear	Clear
Total nucleated cell count, CSF (/uL)	1	0–5
RBC (red blood cell count), CSF (/uL)	2	<1
Neutrophils, CSF (%)	0	0–6
Eosinophil, CSF (%)	0	0
Lymphocyte, CSF (%)	50	40–80
Monocyte, CSF (%)	50	15–45
Macrophages/histiocytes, CSF (%)	0	0
Other cells, CSF (%)	0	0
Glucose, CSF (mg/dL)	92	40–70
Protein, CSF (mg/dL)	48	15–45
CSF culture	No growth 3 days	No growth 3 days
CSF gram stain	No WBCs seen, no organisms seen	No WBCs seen, no organisms seen
Herpes simplex virus 1 PCR, CSF	Negative	Negative
Herpes simplex virus 2 PCR, CSF	Negative	Negative
VDRL, CSF	Negative	Negative

The patient was a poor historian and continued to be disoriented and delirious throughout her hospital stay. She could not identify any sources of exposure and could not recall if she had ever been diagnosed with or treated for syphilis in the past. Her nursing home was also unaware of any potential interactions or exposures that could have led to the patient acquiring syphilis. The patient had been widowed, and her nursing home was not aware of any current partners. Multiple health departments and family members were called to attempt to determine if the patient had been treated for syphilis in the past. No treatment for syphilis in the past could be identified. The patient was diagnosed with latent syphilis but not neurosyphilis and received 2.4 million units of intramuscular penicillin G once a week for three weeks. Psychiatry was consulted for the patient’s delusions and hallucinations. The patient’s new onset delusions, personality change, and hallucinations were attributed to her progressing neurocognitive decline secondary to vascular dementia. Prior to discharge, she continued to have delusions that something was crawling up her legs at night.

## Discussion

As stated before, syphilis is often known as the “great imitator,” and it can be hard to distinguish which manifestations are due to syphilis and which are not. The patient presented with gradual cognitive decline in the past five years, multiple chronic infarctions, dysarthria, extensive cardiac history, new-onset delusions, personality change, and hallucinations. Differential diagnoses included neurosyphilis, progressing neurocognitive decline secondary to vascular dementia, intracranial hemorrhage, meningitis, encephalitis, stroke, cardiac syncope, infectious disease, hypothyroidism, polypharmacy, and delirium secondary to urinary tract infection (UTI), constipation, or other medical condition.

The workup for UTI was negative, and the patient was having regular bowel movements on arrival at the hospital. CT from the previous admission one week earlier and MRI at this admission revealed no acute intracranial abnormalities, ruling out intracranial hemorrhage and stroke. The changes seen on imaging were more consistent with progressive chronic ischemic disease and volume loss. Lumbar puncture with unremarkable CSF findings and negative cultures ruled out meningitis and encephalitis. The echo was negative for cardiac syncope etiology. The TSH was within normal limits during her first admission, and her anticholinergic medications that were thought to be contributing to her previous delirium presentation were discontinued during her first admission. Her other medications were not felt to be contributing to her presentation. HIV studies returned negative. Clinical findings were suggestive of either progressing neurocognitive decline or, once serologic studies were found to be positive for syphilis, possibly latent syphilis with superimposed neurosyphilis.

In this patient, syphilis IGG antibodies returned reactive, suggesting that, unless this was a false positive, the patient had been exposed to syphilis at some time in her life. The patient could not identify any prior syphilis diagnosis or treatment. RPR was negative indicating that there was no tissue damage occurring. If the IGG antibodies were a true positive and if she was truly never treated, then either her infection had been long-standing enough that her titers had declined to a non-detectable state, or her infection was just beginning with no cell damage occurring yet. TP-PA testing was done to reconfirm a positive test and help distinguish between a false positive or true infection. For this patient, the TP-PA antibodies were again positive, confirming a true infection. Consequently, the patient had a confirmed diagnosis of syphilis by two antibody tests. Her test of cell destruction was negative, meaning that she had an early stage of syphilis infection or latent disease. Because the patient, her family, and her nursing home were unaware of any recent exposure to syphilis or any treatment in the past, it was determined that the patient had latent disease. The next step was to determine if her latent syphilis had progressed to neurosyphilis.

The patient had several symptoms suggestive of neurosyphilis: a gradual decline in cognition in the past five years with new-onset delusions, personality change, and hallucinations in the last month; multiple infarctions that could have been caused by meningovascular neurosyphilis; some dysarthria; and intermittent flapping of her hands that was questionable for tremor. Because of these findings, lumbar puncture and CSF VDRL were completed. CSF VDRL, which is highly specific, was negative [5,11]. In addition, the patient’s CSF findings revealed no pleocytosis, and her protein count was only marginally elevated. These results moved neurosyphilis lower on the differential. Her cognitive decline was attributed to her progressive neurocognitive decline secondary to vascular dementia, and she was ultimately discharged with no changes to her initial presentation. The patient was treated only for latent syphilis with three weeks of penicillin intramuscular injections [8].

The question arises about how accurate the CSF VDRL test is and if there are cases that are sometimes negative. Case reports of neurosyphilis with negative CSF VDRL tests have been reported [13]. Further research reveals that a reactive CSF VDRL can establish a diagnosis of neurosyphilis, but a nonreactive test does not exclude the diagnosis. This is secondary to the lack of sensitivity of the CSF VDRL test. CSF VDRL sensitivity is estimated to be only 49-87%, meaning that up to half of patients with neurosyphilis may not test positive [14]. By contrast, the CSF FTA-ABS test is significantly more sensitive for screening despite being less specific with sensitivities of 90.9-100% [5,14]. Due to such a high suspicion of neurosyphilis in this patient and the low sensitivity of the CSF VDRL test, the patient would have benefited from a follow-up CSF FTA-ABS test to rule out this diagnosis more definitively. Because the CSF VDRL is considered the gold standard despite its low sensitivity, a CSF FTA-ABS test was not considered.

## Conclusions

Despite the patient’s negative test results and unremarkable CSF analysis, the patient’s clinical presentation was strongly suggestive of neurosyphilis. Given the strong suspicion of neurosyphilis in this patient, a negative CSF VDRL should have been pursued with a CSF FTA-ABS. A negative CSF FTA-ABS could have excluded the diagnosis of neurosyphilis with more certainty. This case argues that, given the continuously rising rates of syphilis, it would be beneficial to have testing guidelines for neurosyphilis in a similar way to the testing guidelines for syphilis, so suboptimal treatment can be avoided in patients infected with this great imitator.
